# Sex-specific phenotypes of hyperthyroidism and hypothyroidism in aged mice

**DOI:** 10.1186/s13293-017-0159-1

**Published:** 2017-12-22

**Authors:** Helena Rakov, Kathrin Engels, Georg Sebastian Hönes, Klaudia Brix, Josef Köhrle, Lars Christian Moeller, Denise Zwanziger, Dagmar Führer

**Affiliations:** 1 Department of Endocrinology, Diabetology and Metabolism, University Hospital Essen, University Duisburg-Essen, Essen, Germany; 2 Department of Life Sciences and Chemistry, Jacobs University Bremen, Bremen, Germany; 3 Charité-Universitätsmedizin Berlin, corporate member of Freie Universität Berlin,, Humboldt-Universität zu Berlin, and Berlin Institute of Health, Institut für Experimentelle Endokrinologie, Berlin, Germany; 4 Department of Endocrinology, Diabetology and Metabolism, Clinical Chemistry – Division of Laboratory Research, University Hospital Essen, University Duisburg-Essen, Essen, Germany

**Keywords:** Thyroid hormone, Hyperthyroidism, Hypothyroidism, Sex difference, Thyroid dysfunction, Age, Mice, Phenotype

## Abstract

**Background:**

Sex and age play a role in the prevalence of thyroid dysfunction (TD), but their interrelationship for manifestation of hyper- and hypothyroidism is still not well understood. Using a murine model, we asked whether sex impacts the phenotypes of hyper- and hypothyroidism at two life stages.

**Methods:**

Hyper- and hypothyroidism were induced by i.p. T4 or MMI/ClO_4_-/LoI treatment over 7 weeks in 12- and 20-months-old female and male C57BL/6N mice. Control animals underwent PBS treatment (*n* = 7–11 animals/sex/treatment). Animals were investigated for impact of sex on body weight, food and water intake, body temperature, heart rate, behaviour (locomotor activity, motor coordination and strength) and serum thyroid hormone (TH) status.

**Results:**

Distinct sex impact was found in eu- and hyperthyroid mice, while phenotypic traits of hypothyroidism were similar in male and female mice. No sex difference was found in TH status of euthyroid mice; however, T4 treatment resulted in twofold higher TT4, FT4 and FT3 serum concentrations in adult and old females compared to male animals. Hyperthyroid females consistently showed higher locomotor activity and better coordination but more impairment of muscle function by TH excess at adult age. Importantly and in contrast to male mice, adult and old hyperthyroid female mice showed increased body weight. Higher body temperature in female mice was confirmed in all age groups. No sex impact was found on heart rate irrespective of TH status in adult and old mice.

**Conclusions:**

By comparison of male and female mice with TD at two life stages, we found that sex modulates TH action in an organ- and function-specific manner. Sex differences were more pronounced under hyperthyroid conditions. Importantly, sex-specific differences in features of TD in adult and old mice were not conclusively explained by serum TH status in mice.

**Electronic supplementary material:**

The online version of this article (10.1186/s13293-017-0159-1) contains supplementary material, which is available to authorized users.

## Background

Thyroid dysfunction (TD), i.e. hyper- and hypothyroidism, is known to occur with two- to ninefold higher prevalence in women compared to men [1–3], but the influence of sex on clinical manifestation and outcome of TD has not well been studied so far. Interestingly, a recent study indicated that the presence of clinical symptoms is a better predictor for overt hypothyroidism in men and that women report significantly more symptoms after thyroid hormone (TH) replacement therapy than men [4]. Moreover, the risk for atrial fibrillation and reduced bone mineral density appears to be increased in hyperthyroidism in women (at postmenopausal state) compared to men [5].

Data on a possible sex difference in thyroid function parameters such as the thyroid-stimulating hormone (TSH) and serum concentrations of classical THs thyroxine (T4) and triiodothyronine (T3) in cohort studies are inconclusive, with some studies showing sex variance of TH function parameters [6–9] while others do not [10–12]. These contrary findings may be due to differences in cohort characteristics such as age and gender distribution, ethnicity and iodine supply. At the same time, they also illustrate the complexity of TH action in an organism, which may not be directly deducted from serum thyroid function parameters.

In a previous study, we performed a characterization of 5-months-old mice with thyroid dysfunction and identified sex-specific differences in behavioural, functional, metabolic, biochemical and molecular parameters of hyper- and hypothyroidism. Distinctly higher T3/T4 serum concentrations of hyperthyroid female mice did not explain phenotypical differences, as, e.g. no sex impact was noted for heart rate. Moreover differences under control conditions were more frequently preserved with TH excess (body temperature, body weight, nutritional intake), whereas sex impact appeared with TH deprivation for, e.g. cholesterol serum concentrations [13].

**Fig. 1:**
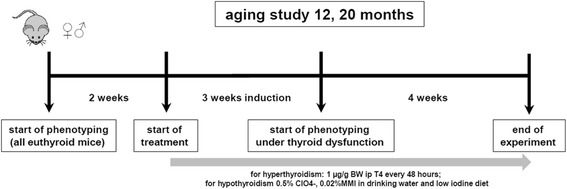
Study design of an approach to identify sex differences of phenotypical traits in thyroid diseases. Aging study was performed to characterize natural sex differences in different life stages between male and female mice in euthyroid, hyperthyroid and hypothyroid conditions [13]

Since TD may occur at any life stage and the prevalence of TD even increases with age, we now asked whether hyper- and hypothyroidism may distinctly impact the male vs. female organism at different ages. By comparing adult (12 months) and old (20 months) with younger (5 months, [13]) mice, we show that age modulates features of TD with more pronounced sex difference under hyperthyroid but less so under hypothyroid conditions. Furthermore, addressing body weight (BW), body temperature, heart rate, activity and neuromuscular function (Fig. 1), we demonstrate a function-specific modulation whereby either sex, THs or age dominates the phenotypic traits.

## Methods

### Animals

For the aging study reported herein, male and female C57BL/6NTac (*n* = 7–11/sex/age/treatment; Taconic Europe A/S, Denmark) mice aged 12 months (adult, *n* = 46) and 20 months (old, *n* = 64) were used. All mice were housed in temperature- (23 ± 1 °C) and light-controlled (inverse 12:12 h light-dark cycle) conditions. Food and water were provided ad libitum. All animal experiments were performed in accordance with the German regulations for Laboratory Animal Science (GVSOLAS) and the European Health Law of the Federation of Laboratory Animal Science Associations (FELASA). The protocols for animal studies were approved by the *Landesamt für Natur*, *Umwelt und Verbraucherschutz Nordrhein-Westfalen* (LANUV-NRW). All efforts were made to minimize suffering.

The 9-week experimental period was divided into three parts, consisting of a 2-week run-in period prior to manipulation of thyroid status to phenotypically characterize each individual mouse (pre-assessment), a 3-week treatment period to induce hyper- or hypothyroidism, and a 4-week assessment period to repeat the phenotypic characterization of each individual animal under chronic TH manipulation or euthyroid control treatment (Fig. 1).

Chronic hyperthyroidism and hypothyroidism were induced as previously described [13–16]. Briefly, for hyperthyroidism, i.p. injections of 1 μg/g body weight T_4_ (Sigma-Aldrich (T2376), USA) were performed every 48 h. For induction of chronic hypothyroidism, animals were fed a low-iodine diet (LoI; TD.95007, Harlan Laboratories, USA) and received drinking water supplemented with 0.02% methimazole (MMI, Sigma-Aldrich (301507), USA), 0.5% sodium perchlorate (ClO_4_^−^) (Sigma-Aldrich (310514), USA) and 0.3% saccharine as sweetener (Sigma-Aldrich (240931), USA) (LoI/MMI/ClO_4_^−^). In addition, hypothyroid animals received i.p. injections of PBS every 48 h. Control animals were fed a control diet (TD.95007 with added potassium iodide (0.0012 g/kg): TD.97350) and received i.p. injections of PBS every 48 h. Body weight was measured three times a week by placing mice on a scale.

### Blood sample collection and analysis of serum TH and TSH concentrations

Final blood samples were obtained by heart puncture in dark phases 24 h after last T4 injection or continuous MMI/ClO4-/LoI treatment and stored 30 min on ice before clearing by centrifugation. Free triiodothyronine (FT3), free thyroxine (FT4) and total T4 (TT4) concentrations in serum of mice were measured using commercial ELISA kits according to the manufacturer’s instructions (DRG Instruments GmbH, Marburg, Germany). Detection ranges were 0.5–25 μg/dL, 0.05–7.5 ng/dL and 0.05–20 pg/mL for TT4, FT4 and FT3, respectively. Serum TSH was measured with a sensitive, heterologous, disequilibrium double-antibody precipitation radioimmunoassay with a detection limit of 10 mU/L [17] (kindly performed by the laboratory of Prof. Refetoff at the University of Chicago, Chicago, USA).

### Measurement of body temperature

Body temperature of mice was assessed four times (approximately 2–4 h after lights were turned off and twice in weeks 6 and 7, respectively) using a cream-covered rectal probe (RET-3 rectal probe for mice, Kent Scientific Corporation, USA) connected to a thermocouple thermometer (Acorn Temp JKT Thermocouple Meter, Kent Scientific Corporation, USA). Mice were placed on the top of the cage and the rectal probe was carefully inserted 2 cm into the rectum until a steady temperature was measured, which took approximately 8 to 10 s.

### Measurement of heart rate

Non-invasive restrained electrocardiography (ECG) recording was performed using an in-house protocol, approximately 3–5 h after lights were turned off [13]. Conscious mice were placed on a platform with their paws on silver electrodes and were restrained by a half-tunnel. Signal was derived, enhanced and digitalized (Picoscope 2204, Pico Technology, United Kingdom). ECG was recorded using Picoscope 6 Software over 60–90 s and heart rate was determined by measurements of RR intervals over 8 s in a stable steady state. Non-invasive restrained ECG was recorded three times in all animals.

### Rotarod test

The rotarod test [13, 18] basically consists of five 3-cm-diameter cylinders, enabling five mice to be tested simultaneously. On the first testing day, mice were allowed to acclimate to the rotarod test by letting them walk 6 min on the rotating cylinder with constant acceleration from 2 to 20 rpm. Experiments were performed in dark phase approximately 3–5 h after lights were turned off.

For each rotarod session, mice were subjected to four trials, with a minimum resting time interval of 6 min between the trials. Rotation mode was switched to constant acceleration from 4 to 50 rpm within 5 min. Maximum time and speed mastered by the animal was recorded. Mice that fell off the rod or attained full speed were placed back to their home cages. Every animal was subjected to two rotarod sessions (with a suspension period of 7 days) each before and after induction of thyroid dysfunction (4 sessions with 16 trials in total). Sessions 2 and 4 were used for statistical analysis.

### Chimney test

The chimney test is constituted of a plastic tube (length 30 cm, diameter 3 cm). Mice were placed inside the tube and allowed to reach the other end. Then the tube was turned into a vertical position with mice head upside down. The test consisted of determining the time taken by mice to climb up to 25 cm of height. Mice were given 90 s of time to pass the test [13, 19, 20]. Chimney test was performed approximately 3–4 h after lights were turned off.

### Open field

The open field consisted of a closed square area made of Plexiglas (50 × 50 cm). The area was divided into four corners, four walls and a center region (16 × 16 cm). Animals were tested in the dark phase of their dark/light cycle, approximately 6 h after lights were turned off. Mice were placed in the center of the open field and allowed to move freely for 5 min. Movements were monitored and digitalized by VideoMot2 software. Software recorded entries in all areas including time, frequency, latency and distance. Occurring events of rearing, freezing, grooming and jumping were recorded manually by the investigator during the experiment. Mice were placed in their home cages after 5 min of exploring the arena.

### Statistical analysis

All data are shown as means ± standard deviation (SD) or standard error of the mean (SEM), as indicated. Statistical analysis using GraphPad Prism 6 Software was performed. The effects of hyper- and hypothyroidism are often opposing, and inclusion of both treatments would therefore always show a significant treatment effect. To prevent false positive results, statistical analyses of treatment groups were performed separately for hyper- and hypothyroid groups. Two-way ANOVA and one-way ANOVA followed by Bonferroni post hoc analysis or unpaired Student’s *t* test were applied as indicated. Values of *p < 0.05, **p < 0.01, ***p < 0.001 were considered statistically significant.

## Results

### Effect of T4 administration on TH serum concentrations is exaggerated in female mice irrespective of age

**Fig. 2:**
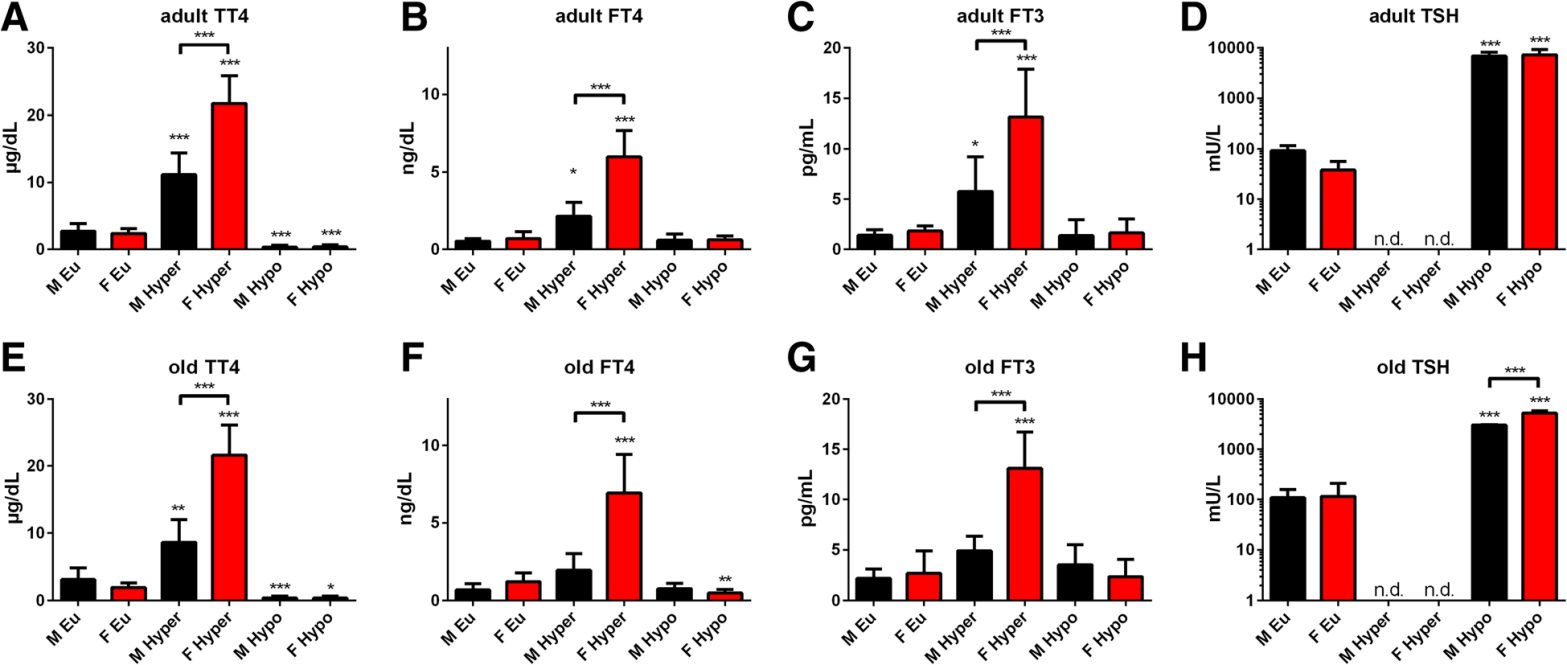
Serum TH status in adult and old male and female mice under TH excess or deprivation. TT4, FT4, FT3 and TSH serum concentrations were determined by ELISA at the end of T4 or MMI/ClO_4−_/LoI treatment in sera of adult (**a–d**) and old (**e–h**) male and female mice. A distinct sex effect on T4 treatment was obvious by higher TH serum concentrations in female than male mice, whereas hypothyroidism resulted in higher TSH elevation in old female mice only. Data are presented as mean ± SD, *n* = 4–11 animals/sex/treatment, two-way ANOVA followed by Bonferroni post hoc analysis for each hyper- and hypothyroid conditions, respectively; *p < 0.05, **p < 0.01, ***p < 0.001; n.d. = not detectable

No significant sex differences were found in euthyroid adult and old mice for TT4, FT4, FT3 and TSH serum concentrations (Fig. 2). Interestingly, T4 treatment resulted in a larger increase of TT4, FT4 and FT3 concentrations in sera of female compared to male mice in adult and even more pronounced in old age (*p* < 0.001, Additional file 1: Table S1). TSH was suppressed as a result of T4 treatment in mice of both sexes and age groups (Fig. 2). TH deprivation resulted in TT4 serum concentrations below assay detection limit (0.5 μg/dL, Fig. 2a, e) and increased TSH in male and female mice (Fig. 2d, h). Free T4 serum concentrations were not altered by TH deprivation except for old female mice showing a reduction of 60% (*F*_(1,30)_ = 5.577 for treatment effect, *p* = 0.0249, Fig. 2f), in line with higher TSH concentrations in these mice (Fig. 2h). Furthermore, MMI/ClO_4−_/LoI treatment did not result in altered FT3 concentrations in male and female mice of both ages (Fig. 2c, g).

### Sex difference in body weight change manifests with T4 treatment and aging

**Fig. 3:**
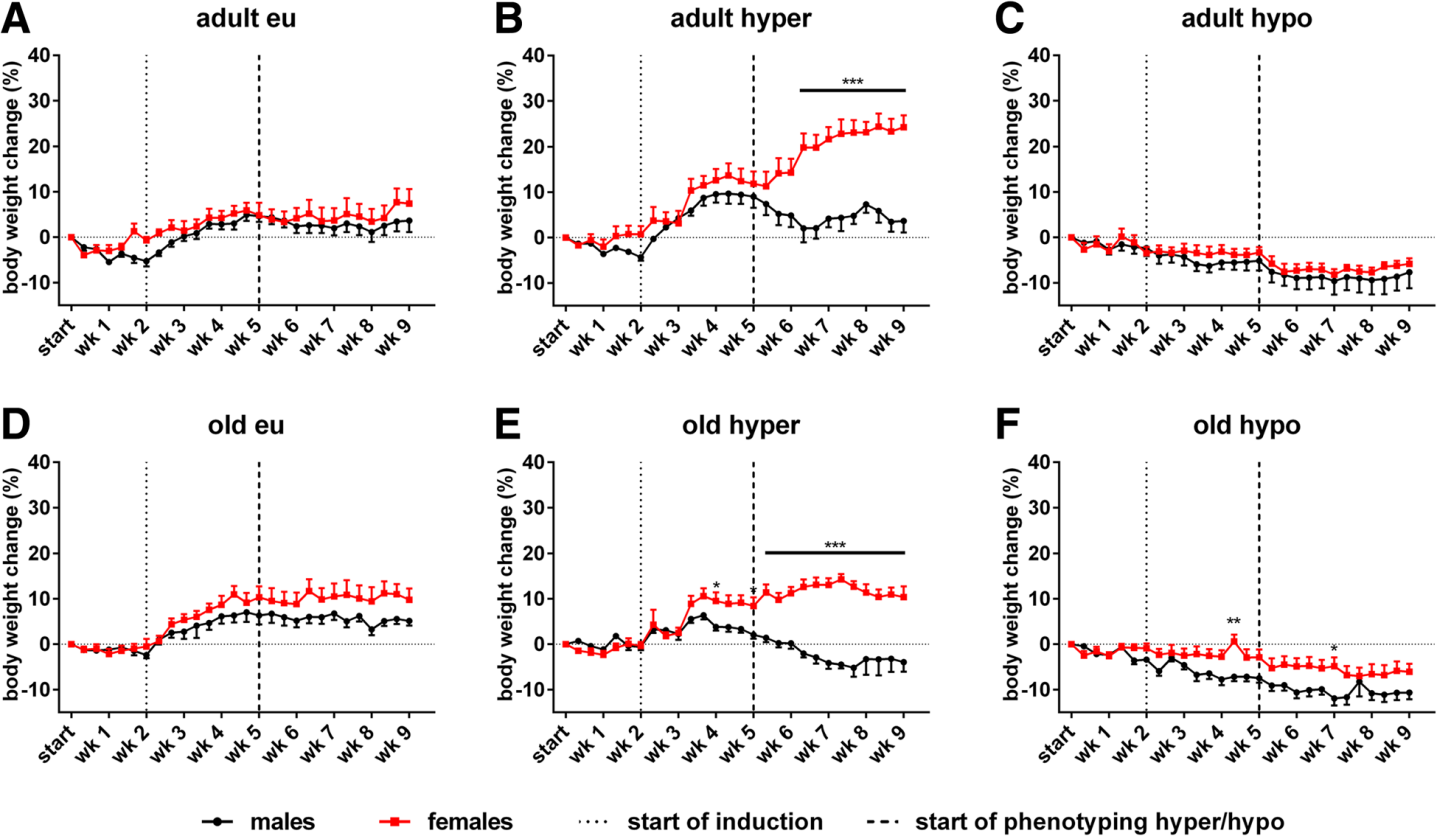
Body weight change of adult and old mice of both sexes in eu-, hyper- and hypothyroid conditions. BW was related to individual start weight in adult (**a–c**) and old (**d–f**) male and female mice. Sex differences in euthyroid (**a, d**), hyperthyroid (**b, e**) and hypothyroid (**c, f**) conditions were assessed. TH excess resulted in BW gain of females, while males lost BW in both ages. No sex difference was observed under TH deprivation. Data are presented as mean ± SD, *n* = 7–11 animals/sex/treatment, two-way ANOVA followed by Bonferroni post hoc analysis, *p < 0.05, **p < 0.01, ***p < 0.001

The impact of thyroid dysfunction on body weight (BW) of male and female mice was assessed over the entire experimental procedure and was compared to the individual body weight at start of experiment (Table 1). Euthyroid male and female adult mice had comparable BW changes throughout the experiment, whereas slightly higher BW increase was noted in old female compared to male mice (Fig. 3a, d, Additional file 1: Table S3). In contrast, T4 treatment resulted in a marked sex difference for BW change. While adult and old females gained weight under TH excess, male mice decreased BW at the same age (for adult: *F*_(27,371)_ = 15.00 for time, *F*_(1,371)_ = 180.2 for sex effect, *F*_(27,371)_ = 5.726 for interaction, *p* < 0.001; for old: *F*_(27,489)_ = 8.01 for time, *F*_(1,489)_ = 353.8 for sex effect, *F*_(127,489)_ = 13.13 for interaction, *p* < 0.001 Fig. 3b, e, Additional file 1: Table S3). In both sexes and age groups, hypothyroidism was accompanied by BW loss, whereby hypothyroid females lost slightly less weight than male mice at old age (Fig. 3c, f). To determine whether the observed BW differences originated from altered nutrient intake, food and water intake were determined weekly and calculated to respective BW of the individual mouse.

**Table 1:** Body weight of adult and old, male and female mice at first (start) and last (final) day of experiment

Age	Sex	Start [g]	Final eu [g]	Final hyper [g]	Final hypo [g]
Adult	Males	40.2 ± 5.0	45.8 ± 3.7 **	40.1 ± 4.1	35.6 ± 2.0 *
	Females	28.0 ± 1.4^††††^	30.5 ± 3.1^††^	34.3 ± 2.2 ***	27.0 ± 1.6
Old	Males	39.0 ± 5.2	41.3 ± 3.7	36.9 ± 5.3	34.0 ± 3.1 *
	Females	30.3 ± 3.5^††††^	32.8 ± 3.2^††††^	33.7 ± 4.4^†^	28.7 ± 1.7^††††^

Data are presented as mean ± SD, *n* = 7–11 animals/sex/treatment, two-way ANOVA followed by Bonferroni post hoc analysis, *p < 0.05, **p < 0.01, ***p < 0.001 multiple comparison to start weight of each sex, ^†^ *p* < 0.05, ^††^ *p* < 0.01, ^††††^ *p* < 0.001 for sex difference at respective thyroid status. *F*_(3,84)_ = 16.07, *p* < 0.0001 for treatment effect in adult, *F*_(3,96)_ = 6.705, *p* = 0.0004 for treatment effect in old mice. *F*_(1,84)_ = 181.6, *p* < 0.0001 for sex effect in adult, *F*_(1,96)_ = 48.68, *p* < 0.0001 for sex effect in old mice and *F*_(3,84)_ = 7.122, *p* = 0.0003 for interaction in adult, *F*_(3,96)_ = 2.078, *p* = 0.1082 for interaction in old mice

While higher food intake in euthyroid female mice was noted in adult and old groups, no difference was found as a result of T4 or MMI/ClO_4_-/LoI treatment (Additional file 2: Figure S1, Additional file 1: Table S3). Similarly, while adult and old euthyroid females consumed more water than male mice, no sex difference was observed under hyperthyroidism at adult age. However, old hyperthyroid females drank notably more water than males. TH deprivation resulted in reversal of sex difference for water intake with male mice consuming more water than females in both ages (Additional file 3: Figure S2, Additional file 1: Table S3).

### Body temperature in contrast to heart rate, is strongly sex-dependent irrespective of TH status and age

**Fig. 4:**
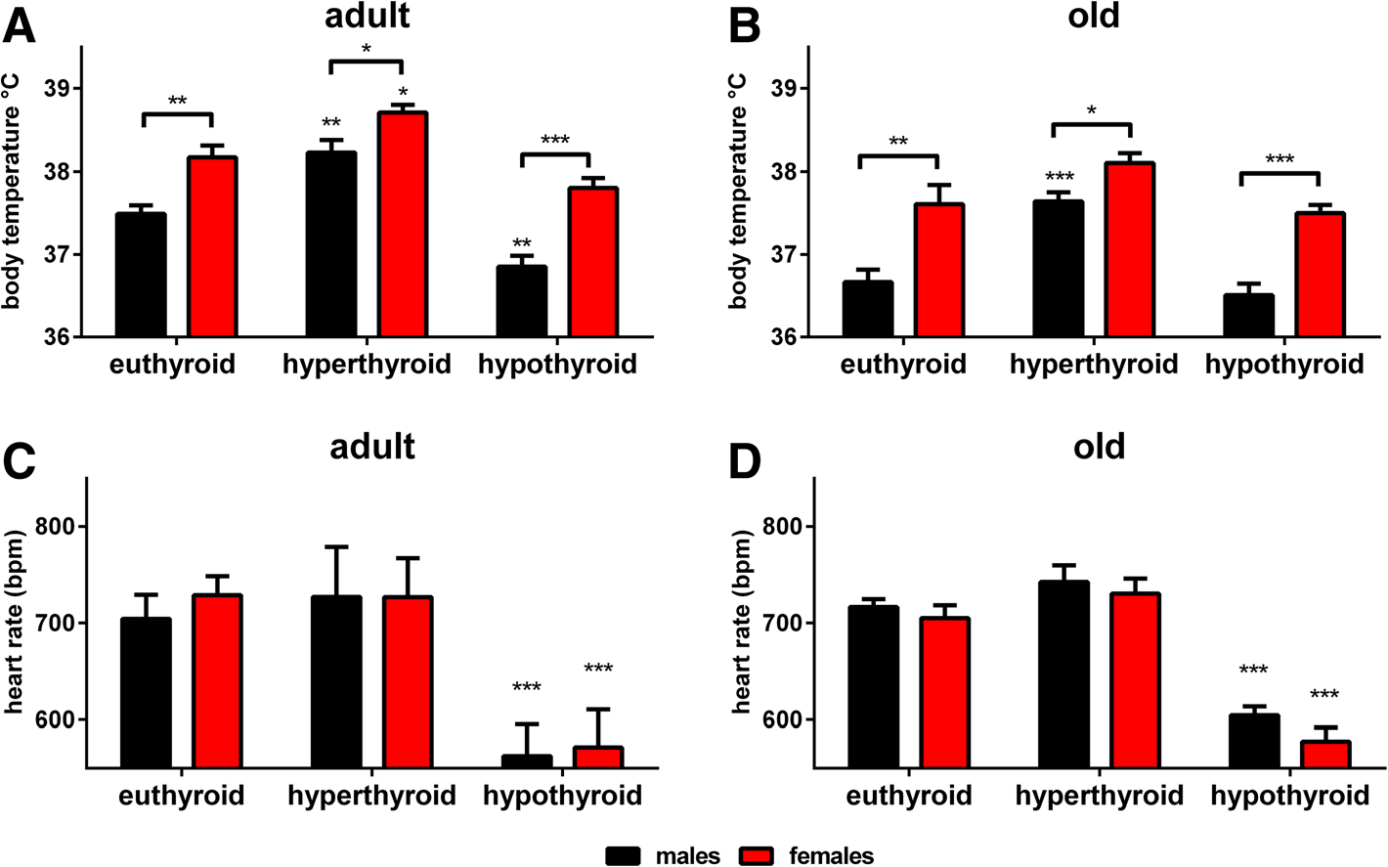
Body temperature and heart rate in adult and old male and female mice with eu-, hyper- and hypothyroid states. Rectal body temperature measurements and non-invasive ECG were performed in adult (**a**) and old (**b**) male and female mice to assess body temperature and (**c, d**) to determine changes in heart rate. Under TH excess and deprivation, persistent higher body temperature of female mice was noted at all TH status and ages, whereas no sex impact was noted for heart rate. Data are presented as mean ± SD, *n* = 7–11 animals/sex/treatment, two-way ANOVA followed by Bonferroni post hoc analysis, *p < 0.05, **p < 0.01, ***p < 0.001

Sex difference in body temperature persisted throughout the entire experimental procedure with a higher body temperature in female compared to male mice in adult and old age and at all TH conditions (Fig. 4a, b, Additional file 1: Table S2).

Non-invasive ECG measurements were performed to examine the influence of sex and TH on heart rate. TH deprivation resulted in a decrease of heart rate in male and female mice, irrespective of age. However, no significant sex differences were found between euthyroid, hyperthyroid and hypothyroid mice at adult and old age (Fig. 4c, d).

### Sex difference on muscle strength and coordination is diminished by TH excess and locomotor activity by TH deprivation in adult mice

To investigate the impact of TD on motor function, muscle strength and activity, male and female mice were subjected to rotarod, chimney and open field tests.

**Fig. 5:**
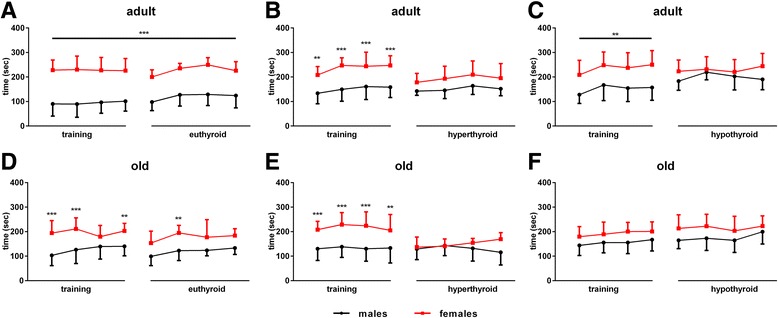
Rotarod performance in all groups in both ages and sexes under control, T4 or MMI/ClO_4−_/LoI treatment. Time spend on the rotating cylinder was determined in adult (**a-c**) and old (**d-f**) male and female mice. For each mouse, a training period was implemented in euthyroid state and was compared to the performance under hyper-, hypo- or again euthyroid state. Pronounced sex difference in euthyroid mice was diminished by TH excess or deprivation. Data are presented as mean ± SD, *n* = 7–11 animals/sex/treatment, unpaired two-tailed Student’s *t* test, *p < 0.05, **p < 0.01, ***p < 0.001

Euthyroid female mice spend more time on the rod compared to male mice at adult and old ages (for adult: *F*_(7,96)_ = 1.680 for time, *F*_(1,96)_ = 279.6 for sex effect, *F*_(7,96)_ = 0.576 for interaction; for old: *F*_(7,120)_ = 1.972 for time, *F*_(1,120)_ = 80.98 for treatment effect, *F*_(7,120)_ = 0.7789 for interaction; Fig. 5a, d), showing better muscle endurance and motor coordination. T4 treatment and TH deprivation resulted in diminished sex differences in adult and old mice with generally poorer performance of hyperthyroid mice and unchanged performance of old hypothyroid mice (Fig. 5b, c, e, f).

**Fig. 6:**
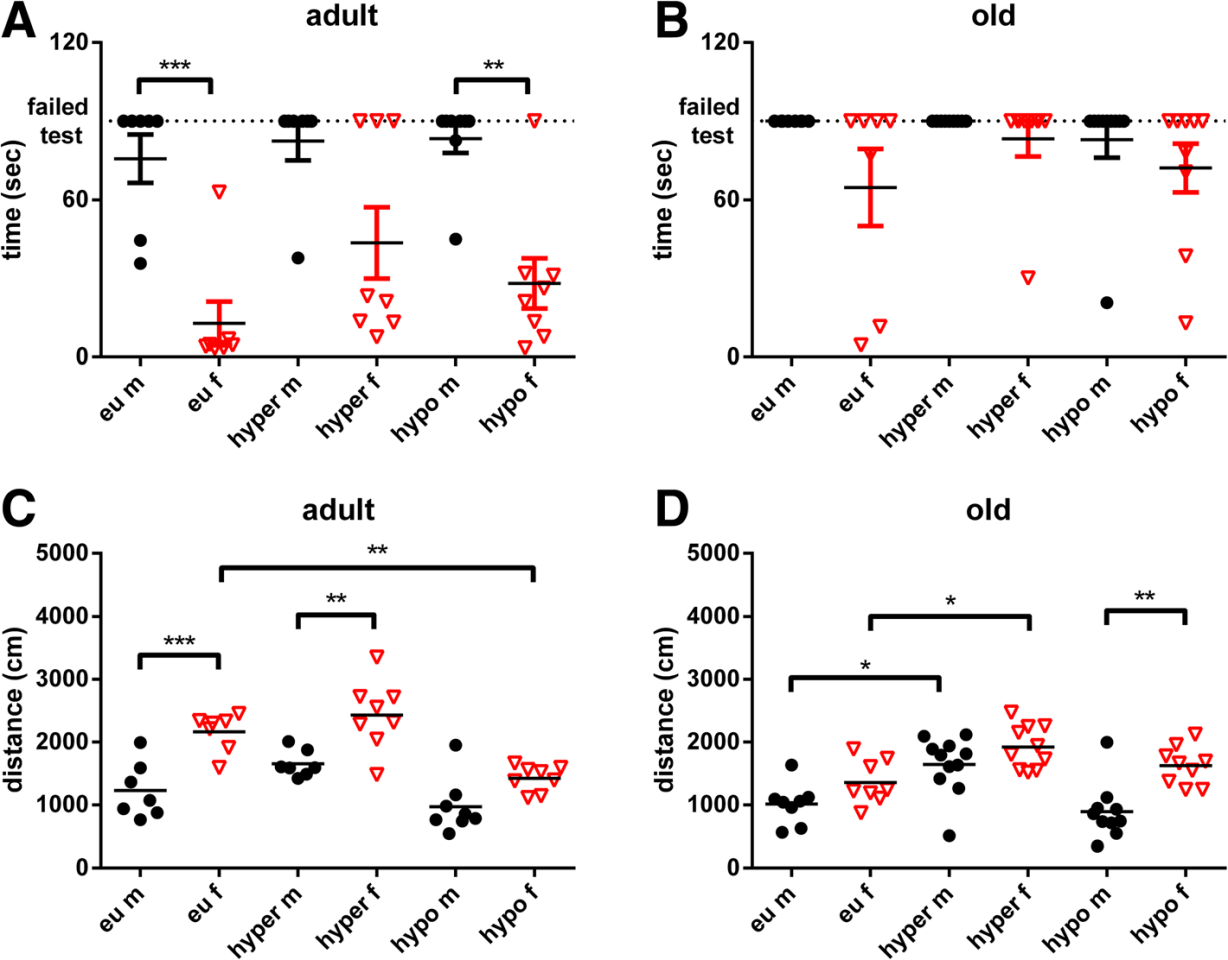
Chimney test and open field activity of adult and old mice of both sexes under eu-, hypo- or hyperthyroid conditions. The Chimney test was performed in adult (**a**) and old (**b**) male and female mice to assess muscle function, while the open field test was performed to assess the activity of mice by measuring the covered distance in adult (**c**) and old (**d**) age under TH excess, deprivation or euthyroid state. Data are presented as mean ± SD, *n* = 7–11 animals/sex/treatment, one-way ANOVA followed by Bonferroni post hoc analysis, *p < 0.05, **p < 0.01, ***p < 0.001

To further evaluate muscle strength, tonus and coordination of movements, we used the chimney test, which measures the required time of mice to climb up in a tube. Overall better coordination of movement and muscle strength was observed in adult female compared to male mice (Fig. 6). Thus, 72% of 12-months-old euthyroid control male mice failed to pass the test, while 100% of female mice succeeded to climb up the tube. This however was impaired under hyper- and hypothyroid conditions with more pronounced impairment as a result of TH excess (failing rate of 37.5% in hyperthyroid, 12.5% in hypothyroid females; *F*_(5,39)_ = 10.13, *p* < 0.001, Fig. 6a). Twenty-months-old male mice were not able to pass the chimney test illustrating the profound effect of age on muscle strength and coordination (Fig. 6b).

Furthermore, locomotor activity was assessed by the open field test. Sex differences were observed at all ages and varied with TH status. In general, higher activity was found in female compared to male mice and this was persistent in eu-, hyper- and hypothyroid state irrespective of age, either as a trend or on a significant level (adult: *F*_(5,39)_ = 16.42, *p* < 0.001, Fig. 6c and old *F*_(5,50)_ = 10.34, *p* < 0.001, Fig. 6d).

## Discussion

Using our animal models of thyroid dysfunction (TD), we could show that sex differences of features of hyper- and hypothyroidism occur throughout mouse life. Thereby, hyperthyroidism was likely to be associated with persistent, exaggerated or new manifestation of sex differences, whereas disappearance of sex effects was observed under hypothyroid conditions. Moreover, phenotypic traits in male and female mice were dominated either by sex (body temperature, muscular strength, locomotor activity), thyroid function (heart rate, muscle endurance and coordination) or age (body weight) and resulted in distinct effects of TD in different organs and functional systems.

### Regulation of body temperature, muscular strength and locomotor activity under TD is dominated by sex

Rectal body temperature differed between younger male and female mice under all TH conditions [13] and persisted in adult and old age with higher body temperature in female than male mice. This was irrespective of thyroid status and suggests a strong sex-dependent effect (Fig. 4a, b). Non-invasive methods of rectal and surface body temperature measurements correlate very well with mice core body temperature [21]. However, a drawback of these methods is that only certain time points can be measured and differences in small time frames may therefore be missed. As reported previously in a study investigating the influence of sex hormones on body temperature via radiotelemetry [22], sex differences were most obvious at daytime and in the proestrous and estrous periods with higher values in 3-months-old females than males. Ovariectomy in these mice resulted in similar body temperature pattern during proestrous and estrous as in male mice, indicating that differences in these time frames were regulated by sex hormones. However, throughout metestrus and diestrus stages, body temperature was comparable between ovariectomized and control female mice suggesting that sex hormones are not the only factors contributing to body temperature regulation in female mice. As no influence of THs was addressed in this study, we conclude from our measurements, that TH excess and deprivation likely have stronger influence on basal body temperature in general, rather than on time points during estrous cycle.

Other examples for the predominant effect of sex on phenotypic traits were muscular strength and locomotor activity. As already described for younger mice [13], a major sex difference was found in the chimney test, which particularly assesses strength and coordination in adult and old mice at eu-, hyper- and hypothyroid mice. Only few old animals were able to pass the test. Hence, conclusions can be derived from adult age only and showed that female mice had a much higher ability to climb up the tube at all TH conditions, while only few male mice were capable to pass the chimney test even at euthyroid state (Fig. 6a). This finding suggests that female mice may have a better movement coordination and muscle strength and are more fatigue resistant than male mice. Sex difference in muscle function has been described in human studies, showing less fatigue and faster recovery in female than male muscles [23] and this observation was also confirmed in a mice study [24]. Interestingly, TD had a stronger impact on females than male mice, with seemingly decrease of sex difference, e.g. in the chimney test in hypothyroid adult mice. While the observed interplay between TH and sex hormones may affect muscle and/or neurological function of the central and peripheral nervous system [25, 26], it may also be influenced by BW and mouse size. As females are lighter and smaller than males at all TH conditions and ages, they may be in a more favourable position to climb up the tube. Thus, when assessing motor function beside muscle strength and endurance, BW and size are likely considerable contributing factors.

Changes in activity were assessed by the open field test and were quantified by measuring the total distance travelled in 5 min. Significant sex difference was noted with higher activity of female mice at different TH conditions and persisted in adult, but differences decreased with old age ([13], Fig. 6c, d). The observed findings are in line with other studies confirming a higher locomotor activity and increased voluntary exercise in female mice [22, 27], indicating an influence of sex hormones on activity of female mice, irrespective of TH status.

### Thyroid function dominates the influence on heart rate, muscle endurance and motor coordination

Another very important issue was the influence of THs and sex on heart rate, as tachycardia or bradycardia are well-known manifestations of TD. Sex difference for heart rate was present in younger euthyroid mice but disappeared by TH excess or deprivation [13], suggesting that alteration of TH status might mask impact of sex hormones. In adult and old mice, no sex impact was found for heart rate, irrespective of TH status (Fig. 4c, d). Thus, TH status dominated the effects of sex and age on heart rate. Of note, measurements of heart rate were performed in a non-invasive ECG, which although mice were pre-trained to the restrainer, may have resulted in increased stress and could have masked small differences in heart rate.

The impact of TD, sex and age on muscle endurance and motor coordination was assessed by the rotarod test. In our previous study, we found that TD impaired performance in young male and female mice with diminished sex difference under hyper- and hypothyroid conditions while euthyroid female mice generally show better results in the rotarod test [13]. In adult and old mice, the sex difference also disappeared under TH excess or deprivation (Fig. 5a–f). Interestingly, this was due to either a weaker performance of female mice (hyperthyroid) or improvement of endurance and motor coordination in males (hypothyroid). Reduced physical function and dexterity has been described in hyperthyroid patients [28], as well as an influence of hypothyroidism on myofiber composition and function [29]. However, to our best knowledge, comprehensive analysis of different motor tests in mice with TD has not yet been performed. Thus, interpretation of our results remains speculative and might not only be a result of changes in muscle function, but could also be dependent on the change of BW due to TD.

### Age modifies sex differences in body weight under TH excess and deprivation

In our initial study, a sex-dependent influence was noted with faster weight gain in hyperthyroid male compared to female mice [13]. In contrast, this sex dependency reversed with age and close to the human situation, adult and old male mice lost BW under T4 excess whereas female mice still gained weight (Fig. 3b, e). Similarly, TH deprivation was accompanied by BW loss in both, male and female mice at adult and old ages, in contrast to unchanged BW in young hypothyroid mice [13]. Hence, both observations implicate that age is a critical factor in BW change under TD in mice, suggesting a switch in metabolism. BW is influenced by energy intake and energy expenditure, the latter depending on physical activity and body temperature. Body temperature itself is a balance between heat production and dissipation [30]. Mice subjected to normal laboratory conditions of 23 °C need approximately 50% of daily food intake to maintain their body temperature whereas clothed humans only need 2% [31] underlining the species-specific traits of metabolism and energy expenditure. Thus, availability of food and amount of food intake is strongly correlated with metabolism and activity [32–34]. Surprisingly, TH excess in young mice resulted in higher food intake in females than males [13]. This sex difference disappeared in hyperthyroid adult and old mice and does not explain the observed differences in BW change of male mice (Additional file 2: Figure S1). Similarly, no significant differences were found for food intake in hypothyroid male and female mice compared to euthyroid controls, suggesting again that food intake is not responsible for BW loss with TH deprivation. Thus, we hypothesize that age-dependent differences in energy expenditure and/or body composition may account for the observed sex-specific BW changes in mice in different life stages.

### Interplay between sex, age and TH status on phenotypical traits of TD is not mirrored in serum TH concentrations

The observed phenotypic traits of TD in male and female mice are not conclusively explained by serum TH concentrations. An obvious difference in response to T4 treatment was observed in all age groups. If subjected to chronic T4 treatment, female but not male mice of all ages had twofold higher TT4, FT4 and FT3 serum concentrations ([13], Fig. 2). This has already been observed by us in an earlier study using young mice [16] and by others using T3 [35], but was not expected to appear in older mice. Different mechanisms may be considered to contribute to this observation. First, T4 injected in the peritoneum needs to be absorbed via blood or lymphatic vessels [36]. Subsequently, within the circulation, the majority of T4 is bound to thyroxin-binding globulin (TBG), transthyretin (TTR) or albumin. Thus, sex dependency in TT4 concentrations could result from differences in binding protein amount or capacity. In mice, however, a higher serum TBG concentration was found in male compared to female mice under euthyroid conditions [37] and was confirmed in our young study on hepatic transcript level [13]. Chronic T4 treatment strongly represses TBG gene expression in both sexes [13] and might therefore unlikely cause different TBG serum concentrations in hyperthyroid male and female mice. However, sex differences on TTR or albumin concentrations might occur under TH excess independent of TBG, but were not investigated in this study.

Strikingly, despite the influence of sex on TT4 serum concentrations and although only a minority of THs circulate in an unbound form, the same sex effect was noted for FT4 and FT3 serum concentrations. Thus, one may speculate that elevated FT4 concentrations in female mice could have resulted from oversaturation of binding proteins, but a conclusive cause remains unclear.

Second, FT4 and FT3 need to be transported into the organs, which are facilitated by specific TH transporters [38]. TH transporter expression on the cell surface can vary with sex, which was shown by us for brown adipose tissue, but less so for liver or heart on a transcript level [13] and could contribute to the observed sex impact on serum concentrations.

Third, as FT3 serum concentrations depend on T4 conversion by deiodinases [39], a sex impact on deiodination also has to be considered. Deiodinase 1 (Dio1) was previously reported to be differently active in young male and female mice. Thereby, renal Dio1 was found to be more active in female mice, while hepatic Dio1 showed higher activity in male than female mice [40]. A subsequent study investigated the effect of age on sexual dimorphism of Dio1, but showed no differences in hepatic Dio1 activity at 12 months of age, while sex differences persisted for renal Dio1 activity [41]. Thus, higher renal Dio1 activity, though not investigated in our study, might have influenced FT3 serum values in female mice.

In contrast, TH deprivation induced by combined MMI/ClO_4−_/LoI treatment, resulted in very low TT4 and unchanged FT3 serum concentrations in all age groups. Thereby, a more severe degree of hypothyroidism occurred in old females as demonstrated by lower FT4 and higher TSH concentrations compared to male mice (Fig. 2).

## Conclusion

In summary, by comparing phenotypic traits of male and female mice with hyper- and hypothyroidism at 5, 12 and 20 months of age, we illustrate that the interplay between sex and age results in distinct effects of hyper- and hypothyroidism on functional systems. While further studies are required to clarify the underlying molecular mechanisms, our data are relevant not only for mice studies addressing either sex, age or TH aspects, but may also be relevant for the clinical setting. Based on our findings, we suggest that further studies are warranted in male and female patients looking at BW and energy expenditure and paying particular attention to neurological and neuromuscular aspects of hyper- and hypothyroidism. Importantly, these studies may also have profound consequences for assessing TD and in the future for considering treatment of TD in women and men at adult and old age.

## Additional files

Additional file 1: Table S1-S3. Statistical analysis of TT4, FT4, FT3 and TSH serum measurements in adult and old mice of both sexes. Two-way ANOVA followed by Bonferroni post hoc analysis was applied. Sex dependency was obvious as shown for Δ(mean female-mean male) values. **Table S2** Statistical analysis of body temperature measurements in adult and old mice of both sexes. Two-way ANOVA followed by Bonferroni post hoc analysis was applied for hyper- and hypothyroid conditions. Average mean values of body temperature are shown as Δ(female-male). **Table S3** Sex differences for area under curve (AUC) analysis of repeated body weight, food and water intake measurements. Two-way ANOVA followed by Bonferroni post hoc analysis was applied to AUC (±SEM) values, calculated by GraphPad Prism 7. (DOCX 18 kb)

Additional file 2: Figure S1. Food intake behaviour during experimental procedure influenced by sex, age and TH condition. Food intake was related to BW weekly in (A-C) adult and (D-F) old male and female mice. Sex dependency was noted for euthyroid groups (for adult: F(8,108) = 24.92 for time, F(1,108) = 430.6 for sex effect, F(8,108) = 9.204 for interaction, *p* < 0.001; for old: F(8,134) = 15.75 for time, F(1,134) = 51.5 for sex effect, F(8,134) = 1.783 for interaction, *p* = 0.0857), which disappeared by TH excess and deprivation. Data are presented as mean ± SD, *n* = 7-11 animals/sex/treatment, 2-way ANOVA followed by Bonferroni post hoc analysis, *p < 0.05, **p < 0.01, ***p < 0.001. (TIFF 743 kb)

Additional file 3: Figure S2. Water consumption in adult and old groups of male and female mice, under control, TH excess or deprivation. Water intake was related to BW weekly in (A-C) adult and (D-F) old male and female mice under euthyroid condition, T4, or MMI/ClO_4−_/LoI treatment. Sex difference was observed in control groups (for adult: F_(8,108)_ = 23.26 for time, F_(1,108)_ = 936.2 for sex effect, F_(8,108)_ = 5.017 for interaction, *p* < 0.001; for old: F_(8,134)_ = 16.38 for time, F_(1,134)_ = 219.4 for sex effect, F_(8,134)_ = 1.788 for interaction, *p* = 0.0847), and was reversed under hyperthyroid adult (F_(8,119)_ = 42.04 for time, F_(1,119)_ = 0.1882 for sex effect, F_(8,119)_ = 13.24 for interaction, *p* < 0.001) and hypothyroid adult and old age (for adult: *F*_(8,126)_ = 15.27 for time, *F*_(1,126)_ = 560.8 for sex effect, *F*_(8,126)_ = 73.19 for interaction, *p* < 0.001; for old: *F*_(8,156)_ = 4.373 for time, *F*_(1,156)_ = 106.0 for sex effect, *F*_(8,156)_ = 9.991 for interaction, *p* < 0.001). Data are presented as mean ± SD, *n* = 7–11 animals/sex/treatment, two-way ANOVA followed by Bonferroni post hoc analysis, *p < 0.05, **p < 0.01, ***p < 0.001. (TIFF 775 kb)

## Abbreviations

BW: Body weight
ClO_4−_: Perchlorate
ECG: Electrocardiography
FT3: Free 3,3′,5-triiodothyronine
FT4: Free thyroxine
LoI: Low-iodine diet
MMI: Methimazole
T_3_: 3,3′,5-triiodothyronine
T_4_: Thyroxine
TD: Thyroid dysfunction
TH: Thyroid hormone
TSH: Thyroid-stimulating hormone
TT_4_: Total thyroxine
