# Supplementary material for: Sex-specific phenotypes of hyperthyroidism and hypothyroidism in aged mice
Source: Biol Sex Differ. 2017 Dec 22;8:38. doi: 10.1186/s13293-017-0159-1 (PMC5741944; doi:10.1186/s13293-017-0159-1)
Supplement: Supplementary file 1 — Statistical analysis of TT4, FT4, FT3 and TSH serum measurements in adult and old mice of both sexes. Two-way ANOVA followed by Bonferroni post hoc analysis was applied. Sex dependency was obvious as shown for Δ(mean female-mean male) values. Table S2 Statistical analysis of body temperature measurements in adult and old mice of both sexes. Two-way ANOVA followed by Bonferroni post hoc analysis was applied for hyper- and hypothyroid conditions. Average mean values of body temperature are shown as Δ(female-male). Table S3 Sex differences for area under curve (AUC) analysis of repeated body weight, food and water intake measurements. Two-way ANOVA followed by Bonferroni post hoc analysis was applied to AUC (±SEM) values, calculated by GraphPad Prism 7. (DOCX 18 kb) [file 13293_2017_159_MOESM1_ESM.docx]

**Supplemental Methods**

# Monitoring of body weight, food and water intake

Food consumption was determined once a week by measuring the weight of remaining food pellets in the metal cage top. Water intake was controlled by weighing of water bottles (each, to the nearest 0.1g) weekly (in control and hyperthyroid groups) or twice (in hypothyroid groups) per week in control and TH manipulated groups. Male mice were caged individually, while female mice were kept in groups of three to four animals. Food and water intake of female mice was calculated by dividing the measured intake by the number of animals in each cage.

For each mouse, average daily food and water intake was calculated, and adjustments for body weight were derived by dividing average intake by the average body weight, and multiplying the result by 40 g (average body weight of mice). Noteworthy, to avoid different nutritional intake, all hypothyroid mouse groups were changed to low iodine diet with start of the protocol for induction of hypothyroidism, while euthyroid and hyperthyroid mice received an equivalent control diet.

**Supplemental table 1:** Statistical analysis of TT4, FT4, FT3 and TSH serum measurements in adult and old mice of both sexes. 2-way ANOVA followed by Bonferroni post hoc analysis was applied. Sex dependency was obvious as shown for Δ(mean female-mean male) values.

| age | adult | old |
| --- | --- | --- |
| Δ(female-male) | TT4=10.6 µg/dL (hyper)  FT4=3.8 ng/dL (hyper)  FT3=7.4 pg/dL (hyper)  TSH=454 mU/L (hypo) | TT4=13.0 µg/dL (hyper)  FT4=5.0 ng/dL (hyper)  FT3=8.2 pg/dL (hyper)  TSH=2188 mU/L (hypo) |
| treatment effect | TT4: *F*_(1,26)_=202.50, p<0.001  FT4: *F*_(1,26)_=92.76, p<0.001  FT3: *F*_(1,26)_=53.67, p<0.001  TSH: *F*_(1,11)_=114.30, p<0.001 | TT4: *F*_(1,26)_=129.70, p<0.001  FT4: *F*_(1,26)_=44.52, p<0.001  FT3: *F*_(1,26)_=60.42, p<0.001  TSH: *F*_(1,11)_=547.50, p<0.001 |
| sex effect | TT4: *F*_(1,26)_=27.40, p<0.001  FT4: *F*_(1,26)_=31.48, p<0.001  FT3: *F*_(1,26)_=13.45, p=0.0011  TSH: *F*_(1,11)_=0.09, p=0.7677 | TT4: *F*_(1,26)_=28.64 p<0.001  FT4: *F*_(1,26)_=27.76 p<0.001  FT3: *F*_(1,26)_=26.79, p<0.001  TSH: *F*_(1,11)_=40.28, p<0.001 |
| interaction | TT4: *F*_(1,26)_=31.01, p<0.001  FT4: *F*_(1,26)_=26.06, p<0.001  FT3: *F*_(1,26)_=10.60, p=0.0031  TSH: *F*_(1,11)_=0.15, p=0.7079 | TT4: *F*_(1,26)_=41.28, p<0.001  FT4: *F*_(1,26)_=17.83, p<0.001  FT3: *F*_(1,26)_=20.90, p<0.001  TSH: *F*_(1,11)_=39.69, p<0.001 |

**Supplemental table 2:** Statistical analysis of body temperature measurements in adult and old mice of both sexes. 2-way ANOVA followed by Bonferroni post hoc analysis was applied for hyper- and hypothyroid conditions. Average mean values of body temperature are shown as Δ(female-male).

| age | adult | old |
| --- | --- | --- |
| Δ(female-male) | eu: 0.68°C  hyper: 0.48°C  hypo: 0.95°C | eu: 0.94°C  hyper: 0.46°C  hypo: 1.00°C |
| treatment effect | hyper: *F*_(1,25)_=28.25, p<0.001  hypo: *F*_(1,26)_=16.48, p<0.001 | hyper: *F*_(1,29)_=22.19, p<0.001  hypo: *F*_(1,31)_=0.719, p=0.403 |
| sex effect | hyper: *F*_(1,25)_=23.43, p<0.001  hypo: *F*_(1,26)_=43.08, p<0.001 | hyper: *F*_(1,29)_=20.13 p<0.001  hypo: *F*_(1,31)_=37.18 p<0.001 |
| interaction | hyper: *F*_(1,25)_=0.707, p=0.408  hypo: *F*_(1,26)_=1.152, p=0.293 | hyper: *F*_(1,29)_=2.326, p=0.138  hypo: *F*_(1,31)_=0.0024, p=0.878 |

**Supplemental table 3:** Sex differences for area under curve (AUC) analysis of repeated body weight, food and water intake measurements. 2-way ANOVA followed by Bonferroni post hoc analysis was applied to AUC (±SEM) values, calculated by GraphPad Prism 7.

| **body weight** | **eu** | **hyper** | **hypo** |
| --- | --- | --- | --- |
| **adult** | AUC=79.4±13.9 (m)  vs. 96.3±21.0 (f)  **n.s.** | AUC=128.2±17.8 (m) vs. 318.6±26.2 (f)  **p<0.001** | AUC=157.8±22.8 (m) vs. 123.5±14.19 (f)  **n.s.** |
| **old** | AUC=112.9±14.8 (m) vs. 187.1±21.8 (f)  **p=0.284** | AUC=69.0±13.0 (m) vs. 206.4±17.6 (f)  **p<0.001** | AUC=187.7±15.1 (m) vs. 94.5±17.9 (f)  **p<0.002** |
| **food intake** | **eu** | **hyper** | **hypo** |
| **adult** | AUC=25.9±0.6 (m) vs. 34.4±0.61 (f)  **p<0.001** | AUC=39.9±0.9 (m) vs. 41.4±0.7 (f)  **n.s.** | AUC=31.9±2.2 (m) vs. 29.5±0.6 (f)  **n.s.** |
| **old** | AUC=30.8±0.85 (m) vs. 34.8±1.0 (f)  **n.s.** | AUC=40.7±1.1 (m) vs. 40.2±1.4 (f)  **n.s.** | AUC=33.1±1.3 (m) vs. 32.3±0.6 (f)  **n.s.** |
| **water intake** | **eu** | **hyper** | **hypo** |
| **adult** | AUC=18.1±0.9 (m) vs. 33.3±0.6 (f)  **p<0.001** | AUC=41.9±1.7 (m) vs. 42.2±0.9 (f)  **n.s.** | AUC=35.8±1.0 (m) vs. 22.5±0.4 (f)  **p<0.001** |
| **old** | AUC=23.4±0.9 (m) vs. 32.4±0.9 (f)  **p<0.001** | AUC=38.6±1.6 (m) vs. 47.2±2.7 (f)  **p<0.001** | AUC=36.9±2.8 (m) vs. 23.4±0.5 (f)  **p<0.001** |

**Suppl. Fig. 1: Food intake behaviour during experimental procedure influenced by sex, age and TH condition.** Food intake was related to BW weekly in (**A-C**) adult and (**D-F**) old male and female mice. Sex-dependency was noted for euthyroid groups (for adult: F_(8,108)_=24.92 for time, F_(1,108)_=430.6 for sex effect, F_(8,108_)=9.204 for interaction, p<0.001; for old: F_(8,134)_=15.75 for time, F_(1,134)_=51.5 for sex effect, F_(8,134)_=1.783 for interaction, p=0.0857), which disappeared by TH excess and deprivation. Data are presented as mean ± SD, n= 7-11 animals/sex/treatment, 2-way ANOVA followed by Bonferroni post hoc analysis, *p<0.05, **p<0.01, ***p<0.001.

**Suppl. Fig. 2: Water consumption in adult and old groups of male and female mice, under control, TH excess or deprivation.** Water intake was related to BW weekly in (**A-C**) adult and (**D-F**) old male and female mice under euthyroid condition, T4, or MMI/ClO_4-_/LoI treatment. Sex difference was observed in control groups (for adult: F_(8,108)_=23.26 for time, F_(1,108)_=936.2 for sex effect, F_(8,108)_=5.017 for interaction, p<0.001; for old: F_(8,134)_=16.38 for time, F_(1,134)_=219.4 for sex effect, F_(8,134)_=1.788 for interaction, p=0.0847), and was reversed under hyperthyroid adult (F_(8,119)_=42.04 for time, F_(1,119)_=0.1882 for sex effect, F_(8,119)_=13.24 for interaction, p<0.001) and hypothyroid adult and old age (for adult: *F*_(8,126)_=15.27 for time, *F*_(1,126)_=560.8 for sex effect, *F*_(8,126)_=73.19 for interaction, p<0.001; for old: *F*_(8,156)_=4.373 for time, *F*_(1,156)_=106.0 for sex effect, *F*_(8,156)_=9.991 for interaction, p<0.001).Data are presented as mean ± SD, n= 7-11 animals/sex/treatment, 2-way ANOVA followed by Bonferroni post hoc analysis, *p<0.05, **p<0.01, ***p<0.001.
