# Supplementary figures and images for: Sex-specific phenotypes of hyperthyroidism and hypothyroidism in aged mice
Source: Biol Sex Differ. 2017 Dec 22;8:38. doi: 10.1186/s13293-017-0159-1 (PMC5741944; doi:10.1186/s13293-017-0159-1)

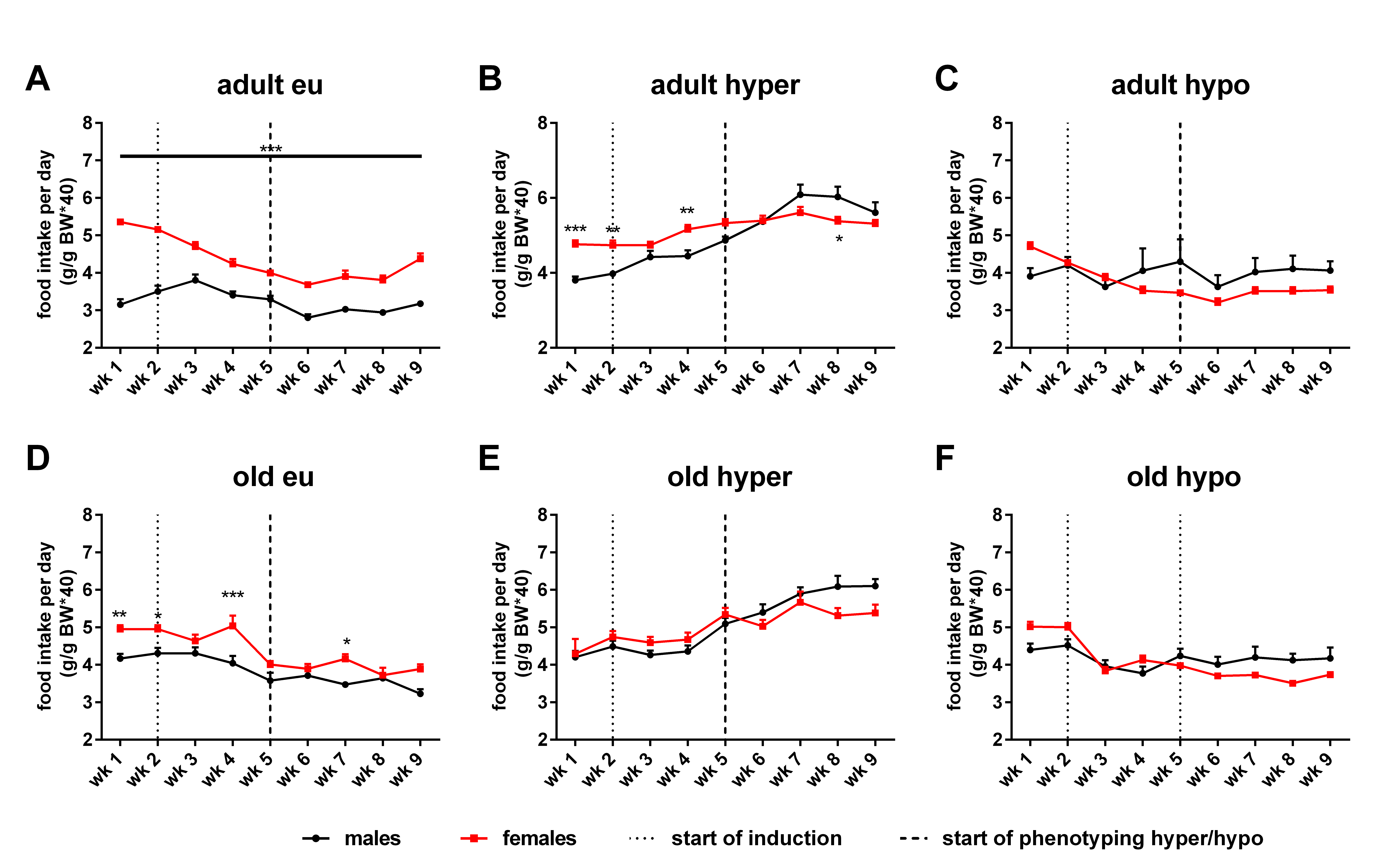

Supplement: Supplementary file 2 — Food intake behaviour during experimental procedure influenced by sex, age and TH condition. Food intake was related to BW weekly in (A-C) adult and (D-F) old male and female mice. Sex dependency was noted for euthyroid groups (for adult: F(8,108) = 24.92 for time, F(1,108) = 430.6 for sex effect, F(8,108) = 9.204 for interaction, p < 0.001; for old: F(8,134) = 15.75 for time, F(1,134) = 51.5 for sex effect, F(8,134) = 1.783 for interaction, p = 0.0857), which disappeared by TH excess and deprivation. Data are presented as mean ± SD, n = 7-11 animals/sex/treatment, 2-way ANOVA followed by Bonferroni post hoc analysis, *p < 0.05, **p < 0.01, ***p < 0.001. (TIFF 743 kb) [file 13293_2017_159_MOESM2_ESM.tif]

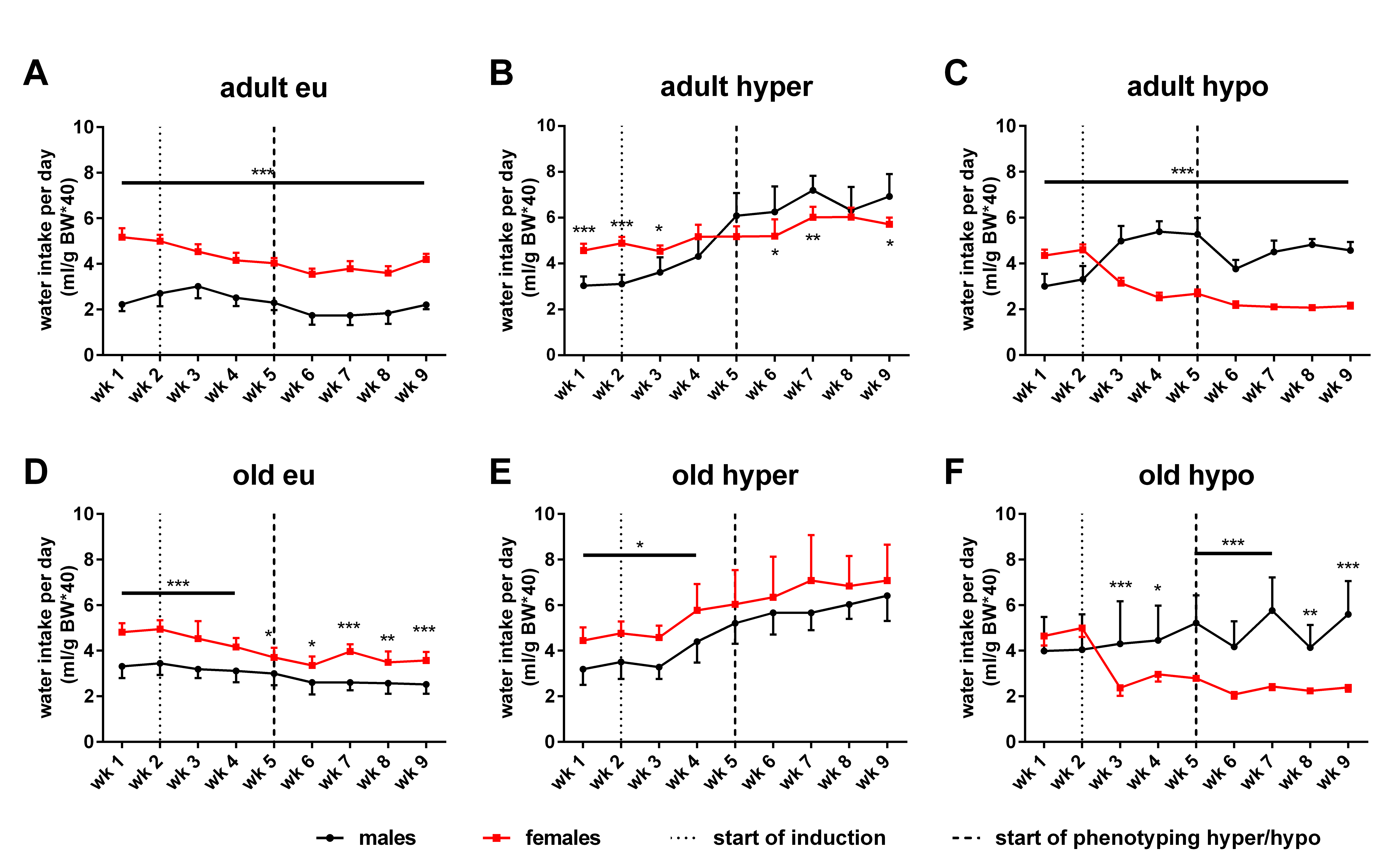

Supplement: Supplementary file 3 — Water consumption in adult and old groups of male and female mice, under control, TH excess or deprivation. Water intake was related to BW weekly in (A-C) adult and (D-F) old male and female mice under euthyroid condition, T4, or MMI/ClO4−/LoI treatment. Sex difference was observed in control groups (for adult: F(8,108) = 23.26 for time, F(1,108) = 936.2 for sex effect, F(8,108) = 5.017 for interaction, p < 0.001; for old: F(8,134) = 16.38 for time, F(1,134) = 219.4 for sex effect, F(8,134) = 1.788 for interaction, p = 0.0847), and was reversed under hyperthyroid adult (F(8,119) = 42.04 for time, F(1,119) = 0.1882 for sex effect, F(8,119) = 13.24 for interaction, p < 0.001) and hypothyroid adult and old age (for adult: F (8,126) = 15.27 for time, F (1,126) = 560.8 for sex effect, F (8,126) = 73.19 for interaction, p < 0.001; for old: F (8,156) = 4.373 for time, F (1,156) = 106.0 for sex effect, F (8,156) = 9.991 for interaction, p < 0.001). Data are presented as mean ± SD, n = 7–11 animals/sex/treatment, two-way ANOVA followed by Bonferroni post hoc analysis, *p < 0.05, **p < 0.01, ***p < 0.001. (TIFF 775 kb) [file 13293_2017_159_MOESM3_ESM.tif]
